# Delphi Consensus on the Role of Venoactive Nutraceuticals in the Management of Chronic Venous Disease: A Position Statement of the Italian Society of Angiology and Vascular Medicine (SIAPAV)

**DOI:** 10.3390/nu17243830

**Published:** 2025-12-07

**Authors:** Giacomo Buso, Paolo Santini, Francesca Ghirardini, Paola Bigolin, Romeo Martini, on behalf of the SIAPAV Working Group on Venoactive Nutraceuticals

**Affiliations:** 1 Department of Clinical and Experimental Sciences, Division of Internal Medicine, ASST Spedali Civili Brescia, University of Brescia, 25123 Brescia, Italy; 2 Faculty of Biology and Medicine, University of Lausanne, 1015 Lausanne, Switzerland; 3 Department of Geriatric, Orthopedic, and Rheumatologic Sciences, Agostino Gemelli University Polyclinic Foundation IRCCS, 00168 Rome, Italy; 4 Faculty of Medicine and Surgery, Catholic University of Sacred Hearth, 00168 Rome, Italy; 5 Angiologia AULSS 1 Dolomiti, San Martino Hospital, 32100 Belluno, Italymartiniromeo@gmail.com (R.M.); 6 UOC Angiologia, Azienda ULSS 2 Marca Trevigiana, 31033 Castelfranco (Treviso), Italy

**Keywords:** chronic venous disease, venoactive nutraceuticals, combination therapy, Delphi consensus

## Abstract

**Background:** Chronic venous disease (CVD) is a prevalent condition associated with significant morbidity and impaired quality of life. Venoactive nutraceuticals are widely used as part of conservative management and are cited in major guidelines, yet recommendations remain heterogeneous and clinical practice varies substantially. This study aimed to establish expert consensus on the clinical use of these agents in CVD within the Italian vascular community. **Methods**: A three-round modified Delphi was conducted among 21 Italian vascular specialists (May–July 2025). Consensus was defined as ≥70% agreement. Statements addressed CEAP classification, symptom assessment, use and perceived effectiveness of individual agents, combination regimens, and topical formulations. **Results**: Consensus supported routine use of CEAP and its 2020 revision, the clinical distinction between CVD (C0–C2) and chronic venous insufficiency (CVI) (C3–C6), and systematic classification of patients as symptomatic or asymptomatic. Strong agreement endorsed the use of selected venoactive nutraceuticals across all CEAP classes and supported combination therapy as more effective than monotherapy. An oral fixed-dose combination of diosmin, ruscus, melilotus, Vitis vinifera, and horse chestnut extract, pre-selected as a test case to evaluate the plausibility of combining agents with complementary mechanisms, was considered a reasonable first-line conservative option. Topical preparations were endorsed across C1–C6, particularly for CVI and to reduce heaviness, reflecting their perceived value as safe adjuncts despite the limited availability of high-quality evidence. No consensus was reached for ruscus, horse chestnut, hydroxyethylrutosides, red vine leaf extract, anthocyanosides, or β-arbutin in monotherapy. The panel agreed on the need to update clinical guidelines to reflect emerging evidence on venoactive nutraceuticals. **Conclusions**: This Delphi provides structured expert consensus on the use of venoactive nutraceuticals in CVD. Combination therapy integrating multiple physiological effects is considered more effective than single agents. Further research is required to validate combination regimens and topical formulations and to determine their impact on patient-reported outcomes.

## 1. Introduction

Chronic venous disease (CVD) is a highly prevalent vascular disorder, affecting between 38 and 90% of adults worldwide and contributing substantially to impaired quality of life (QoL) and healthcare burden [1]. Clinical manifestations range from telangiectasias and reticular veins (C1) to venous ulceration (C6), with symptoms such as heaviness, pain, edema, and skin changes. The CEAP classification remains the cornerstone for diagnosis and staging, and its 2020 revision refined clinical categories and descriptors to improve standardization and clinical applicability [2].

Conventional management strategies for CVD include lifestyle modification, compression therapy, pharmacological treatment, and invasive procedures [3,4,5]. Among pharmacological options, venoactive nutraceuticals are widely used to improve venous tone, microcirculatory function, and symptoms such as edema and heaviness [6]. This heterogeneous group includes diosmin, hesperidin, ruscus extract, horse chestnut seed extract, red vine leaf extract, hydroxyethylrutosides, anthocyanosides, melilotus officinalis, and Vitis vinifera derivatives [5]. Micronized purified flavonoid fraction (MPFF) is a distinct standardized micronized formulation composed of diosmin, hesperidin/diosmetin, linarin, and isorhoifolin. This composition underlies its high bioavailability and clinical efficacy, which supports its endorsement by some international guidelines over single-component agents for the treatment of symptoms and edema in CVD [4,5].

Despite widespread use, guideline recommendations for venoactive nutraceuticals remain heterogeneous, and prescribing practices vary substantially across clinical settings. In Italy, this variability is also evident: venoactive nutraceuticals are widely used across vascular clinics, yet prescribing patterns differ substantially between regions, specialties, and care settings due to heterogeneous training backgrounds, non-uniform product availability, and the absence of nationally harmonized guidance.

To address these inconsistencies, we conducted a Delphi consensus to define the perceived role of venoactive nutraceuticals in the management of CVD within the Italian vascular community. This manuscript, endorsed by the Italian Society of Angiology and Vascular Medicine (*Societa Italiana di Angiologia e Patologia Vascolare*, SIAPAV), presents the consensus findings and discusses their implications, with specific focus on stage-based indications, the relative perceived effectiveness of individual agents, and the rationale and clinical role of combination regimens in routine CVD management.

## 2. Materials and Methods

### 2.1. Study Design and Participants

We conducted a three-round, modified Delphi process between May and July 2025. Only clinicians actively practicing in Italy were eligible, consistent with the study’s aim of generating a nationally coherent consensus. Experts were selected based on: (i) established clinical experience in the diagnosis and management of chronic venous disorders; (ii) active practice in vascular medicine or vascular surgery; and (iii) recognized involvement in the Italian vascular community through clinical activity, scientific contributions, or participation in professional societies. The final Delphi panel included 23 specialists (14 angiologists and 9 vascular surgeons) from 11 Italian regions (Abruzzo, Sardinia, Lazio, Umbria, Marche, Calabria, Campania, Sicily, Veneto, Lombardy, and Liguria), ensuring broad geographical representation across northern, central, and southern Italy, and a balanced mix of hospital-based and community-based settings. No patient-level data were collected; therefore, ethical approval was not required.

This consensus focused exclusively on venoactive nutraceuticals, defined as products derived from food or botanical sources that provide health benefits beyond basic nutrition. This category therefore includes substances such as diosmin, hesperidin, red vine leaf extract, ruscus extract, horse chestnut extract, and other plant-derived flavonoids. The broader term venoactive compounds refers to any substances—nutraceutical or pharmaceutical—shown to exert venotonic, anti-inflammatory, antioxidant, antiedema, or lymphokinetic effects relevant to CVD pathophysiology. By contrast, venoactive drugs denotes pharmaceutical-grade formulations with defined chemical structure, regulatory approval, and standardized manufacturing controls. These differ from nutraceuticals in origin, regulatory framework, and pharmacological development pathway. Accordingly, pharmaceutical agents such as Sulodexide, a glycosaminoglycan not derived from botanical sources, were deliberately excluded. Although MPFF is a registered pharmaceutical product rather than a nutraceutical, it was included because its active constituents originate from botanical sources and are individually classified as nutraceutical substances. Moreover, MPFF represents the most extensively studied venoactive formulation and is consistently evaluated alongside nutraceuticals in international guidelines, making its inclusion essential for a clinically meaningful and comparable assessment. Finally, although various diosmin-based products and flavonoid mixtures exist in different markets, they differ in composition, purity, and degree of micronization, are not considered clinically equivalent to MPFF in regulatory or guideline documents and were therefore not included in this consensus.

### 2.2. Delphi Procedure

Round 1 included exploratory questions addressing: (i) use of CEAP and other assessment tools; (ii) whether and how nutraceuticals should be used across CEAP classes; (iii) perceived effectiveness and safety of individual agents; (iv) the role of combination regimens; and (v) indications for topical preparations. A combination of diosmin, ruscus, melilotus, Vitis vinifera, and horse chestnut extract was included a priori, drawing on expert clinical experience and mechanistic rationale rather than on direct trial evidence, as a test case to assess the plausibility of combining agents with complementary actions for symptomatic CVD.

Responses from Round 1 were both evaluated for agreement and used to generate the structured statements for Rounds 2 and 3. In Rounds 2 and 3, panelists rated each statement on a three-point Likert scale (agree, neutral, disagree). Consensus was predefined as ≥70% agreement.

Items reaching ≥70% agreement in Round 1 proceeded to Round 2 for assessment of consensus stability. If the ≥70% threshold was confirmed in Round 2, the item was not carried forward to Round 3. Items that did not achieve stable consensus in Round 2, either because agreement remained below threshold or because Round 1 consensus did not persist, were re-administered in Round 3. In Round 3, items could: (i) reach consensus for the first time; (ii) confirm consensus (consensus stability); or (iii) fail to reach consensus and be classified as “no consensus”. This approach was adopted to minimize respondent burden while avoiding artificial inflation of consensus.

### 2.3. Outcome Definitions

The primary outcome was the proportion of panelists agreeing with each statement. Secondary outcomes were the direction of change across rounds and thematic insights derived from panel comments. Statements that did not reach consensus were explicitly reported to delineate areas of persisting clinical uncertainty.

## 3. Results

**Figure 1:**
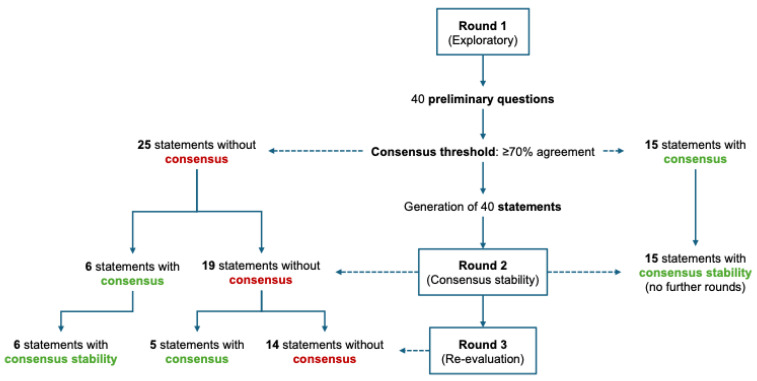
Delphi flowchart.

Overall, the panel reached consensus across the following key domains: classification and assessment, indications for use across CEAP classes, pharmacological strategies encompassing both single agents and combination regimens, and the role of topical formulations. Figure 1 illustrates the structure of the Delphi process whereas Table 1 reports the agreement level obtained in each round for every statement, indicating whether consensus was ultimately reached and in which round. A detailed comparison against major international recommendations provided in Supplementary Table S1.

A total of 21 out of 23 invited specialists completed Round 1. All 21 participants completed Round 2 (0% dropout), and 19 completed Round 3, corresponding to a 9.5% dropout rate across the entire Delphi process. No item was removed due to attrition.

### 3.1. Statements and Commentary

#### 3.1.1. Classification and Assessment

*Statement 1.* CEAP should be used in routine CVD care (agreement 90.5%).

*Statement 2.* The 2020 CEAP revision should be adopted (81%).

*Statement 3.* Distinguishing CVD (C0–C2) from chronic venous insufficiency (CVI) (C3–C6) is clinically relevant (100%).

*Statement 4.* Patients with CVD should be classified as symptomatic vs. asymptomatic (81%).

*Commentary:* Taken together, Statements 1–4 delineate the foundational assessment framework required for consistent decision-making in CVD. All items reached consensus stability by the second round, indicating early and uniform agreement across the panel and reflecting a high level of confidence in these core diagnostic principles. First, using CEAP in routine care aligns directly with major society guidance. Both the latest European Society for Vascular Surgery (ESVS) and the Society for Vascular Surgery, American Venous Forum, and American Vein and Lymphatic Society (SVS/AVF/AVLS) guidelines recommend CEAP—basic/clinical for practice and full CEAP for research—to standardize staging, follow-up, and reporting [3,4]. Adoption of the 2020 CEAP revision is further supported by its practical enhancements, including the “r” modifier for recurrent disease, the explicit inclusion of corona phlebectatica (C4c), and replacement of numeric venous-segment codes with intuitive abbreviations. These changes were developed via a Delphi process to improve reproducibility and usability [2]. Second, distinguishing CVD from CVI has important clinical implications. Contemporary guidance reserves the term CVI for advanced disease stages with edema, skin changes, or ulceration (C3–C6) [7]. This distinction influences diagnostic workup, timing and appropiateness of intervention, and expected outcomes [4,5]. Finally, systematic documentation of symptom status is essential. CEAP incorporates this through “s” and “a” suffixes at every clinical class, reinforcing that treatment decisions should be symptom-driven. This principle is consistent with appropriateness frameworks in clinical practice: for example, the American College of Radiology (ACR) Appropriateness Criteria designate most interventions as “appropriate” only in symptomatic C2–C6 presentations, while procedures for asymptomatic or minimally symptomatic disease are rarely appropriate [8].

#### 3.1.2. Indications for Venoactive Nutraceuticals by CEAP Stage

*Statement 5.* Venoactive nutraceuticals should be used in patients with C0 disease (84.2%).

*Statement 6.* Venoactive nutraceuticals should be used in patients with C1–C2 disease (95.2%).

*Statement 7.* Venoactive nutraceuticals should be used in patients with C3–C6 disease (90.5%).

*Statement 8.* Guideline-recommended venoactive nutraceuticals should be applied routinely in symptomatic CVD management (90.5%).

*Commentary:* The panel endorsed the use of venoactive nutraceuticals across the full CEAP spectrum, with nuances by stage and by agent. Consensus stability for C0 patients was achieved only in the third round, whereas C1–C2, C3–C6, and the use of guideline-supported agents reached the threshold in the first two rounds. This pattern suggests initially lower confidence in treating C0 disease, followed by convergence after iterative review, while consensus for all other stages was already stable and uniform. Clinical guidance supports this graded interpretation. For C0s, the International Union of Angiology (IUA) recommends pharmacologic therapy for symptom relief. MPFF carries a strong, moderate-to-high quality recommendation for alleviating CVD symptoms from C0s through C6s [5], and large observational data from the RELIEF study demonstrated clinically meaningful improvement in pain, cramps, edema, QoL, and a shift toward lower CEAP classes after 6 months of treatment [9]. These findings reinforce that the rationale for treatment in C0 is symptom-driven rather than strictly stage-dependent. From C1 onwards, the evidence base is more robust. The SVS/AVF/AVLS 2023 guidelines suggest using venoactive drugs for symptomatic varicose veins in patients who are not candidates for intervention, are awaiting intervention, or continue to have symptoms after intervention, with the strength of recommendation maintained but the evidence level varying by agent [4]. Similarly, the ESVS 2022 guidelines advise considering venoactive drugs for symptomatic CVD and/or edema regardless of planned or completed venous interventions, while emphasizing the importance of selecting agents based on their specific pharmacologic actions [3]. In this respect, the IUA guidelines provide even more granular recommendations, ranking MPFF, ruscus-based combinations, and horse chestnut extract among the agents with the strongest evidence for symptomatic relief, although with variation among individual symptoms [5]. The panel’s strong agreement that guideline-endorsed venoactive nutraceuticals should be used routinely in symptomatic CVD reflects this approach: prioritize agents with the most robust evidence for the patient’s clinical presentation, integrate them with compression and lifestyle measures, and periodically reassess benefit. At the same time, recent guidelines have tended to issue weaker recommendations for most single agents. Since individual nutraceuticals may target only specific mechanisms or symptom clusters, rational combination therapy may help broaden both symptomatic and pathophysiological coverage.

#### 3.1.3. Individual Agents

*Statement 9–10.* MPFF should be used and is effective for symptoms improvement (90.5% and 85.7%, respectively).

*Statement 11–12.* Diosmin should be used and is effective for symptoms improvement (95.2% and 100%, respectively).

*Statement 13–14.* Hesperidin should be used and is effective for symptoms improvement (89.5% and 73.7%, respectively).

*Statement 15.* Clinical guidelines should be updated to reflect the role of such nutraceuticals in CVD treatment based on new evidence (85.7%).

*Commentary:* The panel’s endorsement for MPFF, diosmin, and hesperidin reflects the strength and distribution of the current evidence base. Consensus for MPFF and for the need to update guidelines was reached in the first two rounds, whereas one of the diosmin statements and both hesperidin statements required a third round to reach stability, indicating slightly lower initial confidence for hesperidin used as a single agent. MPFF is supported by consistent randomized evidence demonstrating symptomatic and anti-edema effects. On quantitative analysis, a systematic review and meta-analysis of 7 placebo-controlled randomized controlled trials (RCTs) (*n* = 1692) showed significant improvements in pain, heaviness, swelling sensation, cramps, paresthesia, pruritus, functional discomfort, QoL, and ankle circumference [10]. Real-world data from the RELIEF study also documented substantial clinical improvements and even shifts toward lower CEAP classes over six months of treatment [9]. MPFF is a micronized formulation in which diosmin accounts for about 90% of the active fraction, withthe remaining ~10% composed of hesperidin derivates. Diosmin received agreement as both a treatment option and an effective agent, thus emerging as a valid alternative to MPFF. This aligns with guideline assessments and comparative evidence. Meta-analytic data indicate that MPFF provides among the strongest available support for symptom and edema reduction, but diosmin monotherapy is also beneficial [4]. A review of RCTs comparing diosmin and MPFF reported ~50% symptom reductions over 1–6 months without significant differences between agents [11]. Furthermore, a non-inferiority RCT comparing non-micronized diosmin 600 mg and micronized diosmin 900 mg plus hesperidin 100 mg in C0s–C3 patients demonstrated non-inferior efficacy, with the non-micronized formulation showing greater ease in swallowing the tablet [12]. Hesperidin was endorsed more modestly, consistent with its evidence base. High-quality trials evaluating hesperidin alone are limited, and much of its supportive evidence derives from combination formulations rather than monotherapy. This likely explains why agreement on “effectiveness” (73.7%) was the lowest among the major flavonoids, even though its use (89.5%) was widely supported.

Finally, the strong consensus on the need to update clinical guidelines reflects the acknowledgement that emerging evidence on nutraceuticals has outpaced current recommendations. While current guidance mainly rely on a few established single agents, future research high-quality trials assessing fixed-dose combinations with complementary mechanisms of action may further shape individualized therapeutic strategies and broaden the range of drugs supported by guidelines.

#### 3.1.4. Combination Regimens

*Statement 16.* A combination of venoactive nutraceuticals should be used for symptomatic CVD (100%).

*Statement 17.* Combination therapy is more effective than monotherapy (90.5%).

*Statement 18.* An oral combination of diosmin, ruscus, melilotus, Vitis vinifera, and horse chestnut extract is a reasonable first-line conservative option (85.7%).

*Commentary:* The panel strongly endorsed combination therapy, reflecting the view that individual nutraceuticals target multiple pathophysiological mechanisms, so that combining agents may broaden symptomatic coverage compared with monotherapy. All three statements in this domain reached consensus across the first two rounds, indicating early and stable agreement regarding the perceived clinical value of multimodal regimens. This perspective is consistent with the IUA consensus, which maps venoactive substances according to their venotonic, anti-inflammatory/antioxidant, anti-edema, and lymphokinetic actions, thereby providing a mechanistic rationale for combining agents when patients present with clustered symptoms such as heaviness, pain, edema, and skin changes [5]. The most robust evidence for fixed combinations derives from studies on ruscus + hesperidin methyl chalcone + vitamin C, which improved pain/heaviness and reduced ankle circumference compared with placebo or active comparators in randomized trials [13]. The IUA guidelines therefore give strong support to this combination for the control of symptoms and edema [5]. Additional supportive evidence exists for other components. Vitis vinifera extracts have demonstrated improvements in microvascular parameters and limb volume in RCTs [14,15], with more recent data suggesting symptomatic non-inferiority to MPFF in selected settings [16]. Melilotus has smaller but positive studies showing benefits on symptoms and edema [17]. Horse chestnut extract meta-analyses have shown reduction in pain and leg volume, albeit with greater heterogeneity compared with MPFF [18]. Taken together, these strands justify the panel’s view that a multimodal combination including diosmin, ruscus, melilotus, Vitis vinifera, and horse chestnut extract may be considered a reasonable first-line conservative option when multiple symptom domains or mechanisms are clinically relevant. At the same time, it is important to emphasize that no RCT has evaluated this exact five-agent combination, and therefore the panel’s endorsement reflects expert opinion based on mechanistic plausibility and extrapolated evidence, rather than trial-proven superiority over monotherapy. Accordingly, we underscore that such combinations should be used with periodic reassessment of clinical benefit and tolerability, pending more robust evidence to substantiate their role in CVD.

#### 3.1.5. Topical Formulations

*Statement 19.* Topical agents should be used in C1–C2 disease (78.9%).

*Statement 20.* Topical agents should be used in C3–C6 disease (84.2%).

*Statements 21–25.* Targets endorsed by topical agents should include improvement of edema and hematoma (94.7%), heaviness (84.2%), itching/burning (84.2%), skin changes (73.7%), as well as their combination (84.2%).

*Statement 26.* A topical combination of diosmin, ruscus, melilotus, Vitis vinifera, and horse chestnut extract is a reasonable first-line conservative option (90.5%).

*Commentary:* The panel’s endorsement of topical therapy across C1–C6 aligns with the guideline view of conservative care as modular and combinable: in symptomatic CVD, measures such as compression, lifestyle changes, and venoactive agents may be used alone or together, tailored to the symptom cluster and reassessed over time [3]. Within this framework, topical agents are considered pragmatic adjuncts, particularly when localized symptoms persist despite systemic therapy or interventions. However, consensus in this domain showed greater heterogeneous than for oral agents.

As detailed in Table 1, no consensus was reached for the use of topical agents in C0 disease. The indication for C1–C2 required three rounds to achieve agreement, while C3–C6 reached consensus in both Rounds 2 and 3. Among symptom-specific targets, reduction in edema and hematoma, itching/burning, skin changes, and all combined targets achieved consensus only in Round 3, whereas heaviness relief was confirmed across Rounds 2 and 3. By contrast, the predefined five-component topical combination reached the consensus threshold already in Round 2 and therefore did not progress to the third round. This pattern suggests more variable clinical confidence and experience with topical therapy, likely reflecting the comparatively limited evidence base. Pre-clinical studies indicate venotonic and microvascular-permeability effect for flavonoids (e.g., diosmin) and horse chestnut extract, and veno-kinetic activity for ruscus in healthy volunteers [19,20,21]. These findings provide biological plausibility for edema control and symptom relief when applied locally. Evidence for hematoma reduction is limited but mechanistically consistent with capillary-stabilizing actions [5]. Robust clinical trial data remain lacking.

As in the oral domain, the five-component topical combination had been predefined to assess the plausibility of a multimodal, mechanism-based approach. This item reached consensus early, reflecting consistent and stable agreement on the value of combining agents with complementary mechanisms even within an area where supporting evidence is less robust. It is important to note that the panel’s endorsement of topical agents, particularly in C3–C6, draws primarily from clinical experience and mechanistic plausibility rather than from high-quality randomized evidence, which remains scarce in advanced CVD. Further well-designed clinical trials are needed in this regard.

#### 3.1.6. Statements Without Consensus

*Statement 27.* Validated QoL questionnaires (generic and disease-specific) should be used before and after treatment to assess CVD symptoms (47.4%).

*Commentary:* A plausible interpretation of the panel’s lack of consensus regarding the routine administration of validated QoL questionnaires is the persistent gap between guideline-level advocacy for patient-reported outcome measures (PROMs) and the operational feasibility of their use in everyday clinics. The ESVS 2022 guideline considers PROMs central to assessing treatment success and lists validated disease-specific instruments (e.g., AVVQ, CIVIQ, VEINES-QOL/Sym), yet acknowledges that they are time-consuming and infrequently implemented outside trials and structured registries [3]. Similarly, the SVS/AVF/AVLS 2023 document recommends the revised Venous Clinical Severity Score (r-VCSS) for routine grading and follow-up, partly because it correlates with PROMs while being more pragmatical at the point of care [4]. The IUA consensus likewise catalogs both generic and disease-specific QoL tools and documents QoL gains with effective conservative measures [5]. In practice, these sources suggest a balanced interpretation: QoL tools are valuable and aligned with best practice, but real-world constraints limit routine adoption. A pragmatic pathway might be to ensure baseline and follow-up symptom capture for all patients (CEAP + r-VCSS), integrating a brief validated PROM at key decision points when feasible.

*Statement 28–29.* Ruscus should be used and is effective for symptoms improvement (52.6% each).

*Statement 30–31.* Horse chestnut extract should be used and is effective for symptoms improvement (57.9% each).

*Statement 32–33.* Hydroxyethylrutosides should be used and is effective for symptoms improvement (61.1% and 42.1%, respectively).

*Statement 34–35.* Red vine leaf extract should be used and is effective for symptoms improvement (26.3% and 21.1%, respectively).

*Statement 36–37.* Anthocyanosides should be used and is effective for symptoms improvement (63.2% and 42.1%, respectively).

*Statement 38–39.* β-arbutin should be used and is effective for symptoms improvement (26.3% each).

*Commentary:* In keeping with contemporary guideline appraisals, the panel’s hesitant endorsement of several venoactive nutraceuticals reflects the heterogeneous and generally low-to-moderate quality of the available evidence. For ruscus, both ESVS 2022 and SVS/AVF/AVLS 2023 guidelines summarize small, heterogeneous double-blind RCTs showing improvements in limb symptoms and objective edema, but methodological limitations temper confidence [3,4]. Horse chestnut seed extract has more substantial evidence, including a Cochrane meta-analysis and additional RCTs demonstrating superiority over placebo for leg pain and volume, with one study suggesting non-inferiority to compression for selected outcomes [18,22]. However, trial heterogeneity and risk of bias result in inly moderate endorsements in guidelines and modest agreement within the panel. Hydroxyethylrutosides show broadly positive effects in older RCTs and a systematic review [23,24], yet inconsistent dosing and variable preparations lead major guidelines to issue only weak recommendations [3,5]. Red vine leaf extract is supported by several placebo-controlled trials demonstrating improvements in microvascular parameters, ankle/calf circumference and leg volume [14,15,25], with a recent systematic review confirming symptomatic benefit [26]. Nonetheless, effect sizes are modest and study designs heterogeneous, which likely explains the panel’s very low endorsement for as a standalone therapy. Evidence for anthocyanosides is even more limited and largely extrapolated from the red-vine-leaf studies. Guidelines list the class but do not provide strong recommendations [3,4,5]. Finally, β-arbutin is not addressed as a venoactive agent by major guidelines, and no robust evidence supports its use for symptom relief in CVD. Overall, the failure to reachconsensus for these agents should be interpreted not as neutrality but as a clear indicator of clinical uncertainty. The uneven evidence base, together with variability in extract standardization and the absence of high-quality comparative trials, parallels the cautious stance of international guidelines. These findings delineate explicit priorities for future well-designed randomized studies using standardized formulations and harmonized outcome measures.

*Statement 40.* Topical agents should be used in C0 disease (22.2%).

*Commentary:* The panel’s low agreement regarding topical therapy in C0 patients aligns with the limited evidence available for this specific subgroup. Although small studies of multi-ingredient topical formulations (e.g., melilotus-containing creams) have reported short-term improvements in heaviness, pain, itch/burning, swelling and skin hydration [27], these investigations typically enrolled symptomatic CVD patients without distinguishing pure C0 presentations. Consequently, there is insufficient stratified evidence to support routine use of topical agents in individuals with symptoms but no visible clinical signs, and extrapolation from broader symptomatic cohorts remains uncertain.

## 4. Discussion

This Delphi provides a concise, practice-oriented framework for the use of venoactive nutraceuticals in CVD within the Italian vascular community. Unlike previous guideline documents, which largely focus on individual agents, our findings capture how expert practice has evolved toward a multimodal, mechanism-based approach.

The panel’s positions were broadly aligned with ESVS 2022 and SVS/AVF/AVLS 2023 guidelines, while adding granularity regarding perceived effectiveness, clinical contexts of use across CEAP classes, symptom-specific targets, and the role of combination strategies, areas where existing guidelines remain limited or generic. The strongest consensus emerged for combination therapy, reflecting the complementary physiological actions of different nutraceuticals and the need to address multiple symptom domains simultaneously. This perspective is consistent with the mechanistic rationale outlined in the IUA consensus, but it has not yet been explicitly incorporated into major guideline recommendations. Equally important, the lack of consensus for several commonly used agents, such as ruscus, horse chestnut, hydroxyethylrutosides, and anthocyanins, should be regarded as a substantive finding. This reflects the heterogeneous and often low-certainty evidence supporting these agents, as well as variability in extract standardization and the absence of high-quality comparative trials. These gaps identify clear research priorities that warrant rigorous, formulation-standardized RCTs.

Our study has several limitations. First, it relies on expert opinion rather than direct patient-reported outcomes or objective clinical endpoints. While appropriate for addressing practice variability in areas where evidence is incomplete, future research integrating patient-centered data (including PROMs, QoL measures, and objective outcome metrics) will be crucial to validate and refine these recommendations. Second, the exclusive inclusion of Italian specialists inherently limits the generalizability of our findings to other healthcare systems. This was an intentional methodological choice, as the primary aim of the Delphi was to address the marked heterogeneity in nutraceutical prescribing currently observed within the Italian vascular community. Nevertheless, we acknowledge that prescribing practices, product availability, and regulatory environments differ internationally, and future Delphi efforts, including multinational panels, will be essential to validate and extend these recommendations. Third, the statements are intended to complement, not replace, evidence-based guideline recommendations. Areas where consensus was not reached highlight clinical uncertainty and should delineate future research directions, including comparative trials, evaluations of standardized fixed-dose combinations, and studies incorporating PROMs. Similarly, while the panel expressed strong support for topical agents, especially in C3–C6 disease, this position reflects pragmatic clinical reasoning and mechanistic plausibility rather than high-level evidence, underscoring the need for dedicated RCTs in advanced disease. Finally, cross-country epidemiological data on nutraceutical use in CVD remain limited due to heterogeneous regulatory classification. This variability was a key motivation for conducting the consensus, which aims to reduce educational gaps that persist despite existing societal recommendations.

The limited and inconsistent evidence supporting many venoactive nutraceuticals also contributes to their frequent exclusion from insurance coverage and government reimbursement schemes, restricting patient access. Although reimbursement was outside the scope of this Delphi, future policy-oriented work will be necessary to address the ethical and economic implications of coverage, particularly for patients with advanced CVD.

Future efforts should also focus on developing practical, point-of-care tools to facilitate implementation of conservative strategies in CVD management. Translating consensus recommendations into clear clinical algorithms and flowcharts may improve consistency across care settings. Targeted educational initiatives, including focused symposia, could reinforce the multimodal approach and ensure that conservative therapies, such as compression, lifestyle modification, and venoactive nutraceuticals, are fully integrated into routine practice.

Finally, emerging evidence suggests that genetic variability may significantly influence the metabolism and vascular effects of flavonoid-based nutraceuticals. Polymorphisms in enzymes and transporters involved in (poly)phenol metabolism, such as COMT, UGTs, SULTs, and ABC efflux transporters, can modulate bioavailability and physiological responses [28,29]. Although pharmacogenetic considerations were beyond the scope of this Delphi, these findings represent an important avenue for future research. Studies evaluating whether genetic polymorphisms modify responses to venoactive nutraceuticals could lay the groundwork for more personalized, genotype-informed therapeutic strategies in CVD.

## 5. Conclusions

Through a structured Delphi process, we established consensus on key aspects of venoactive nutraceutical use in CVD. Combination therapy emerged as the preferred strategy, reflecting the complementary mechanisms of different agents, while diosmin, MPFF, and hesperidin were endorsed as reliable single-agent options. Topical formulations were deemed reasonable, particularly across C3–C6 classes, for addressing symptoms such as limb heaviness, although supporting clinical evidence remains limited. Areas where consensus was not achieved point to clear evidence gaps, particularly regarding the clinical effectiveness of several individual venoactive nutraceuticals with heterogeneous or limited data, the routine integration of PROMs into everyday practice, and the role of topical therapies in early disease stages. Overall, these consensus statements provide a pragmatic framework to support more consistent clinical practice and to guide future updates to evidence-based recommendations.

## Figures and Tables

**Table 1:** Consensus Summary Table.

Domain	Statement	Agreement (%)			
		1st Round (*N* = 21)	2nd Round (*N* = 21)	3rd Round (*N* = 19)	Consensus (≥70%)
Classification and assessment	Use of CEAP classification in routine CVD care	81.0	90.5	–	Yes
	Adoption of 2020 revision of the CEAP classification	76.2	81.0	–	Yes
	Clinical distinction between CVD (C0–C2) from CVI (C3–C6)	85.7	100.0	–	Yes
	Classification of patients as symptomatic vs. asymptomatic	76.2	81.0	–	Yes
	Use of validated QoL questionnaires in routine practice	14.3	52.4	47.4	No
Indications across CEAP classes	Use of venoactive nutraceuticals in C0	66.7	71.4	84.2	Yes
	Use of venoactive nutraceuticals in C1–C2	81.0	95.2	–	Yes
	Use of venoactive nutraceuticals in C3–C6	90.5	90.5	–	Yes
	Use of guideline-endorsed venoactives in symptomatic CVD	85.7	90.5	–	Yes
Single-agent selection	MPFF to be used in CVD	85.7	90.5	–	Yes
	MPFF considered effective in improving symptoms of CVD	76.2	85.7	–	Yes
	Diosmin to be used in CVD	71.5	95.2	–	Yes
	Diosmin considered effective in improving symptoms of CVD	66.7	95.2	100.0	Yes
	Ruscus extract to be used in CVD	28.6	47.6	52.6	No
	Ruscus extract considered effective in improving symptoms of CVD	33.4	57.1	52.6	No
	Horse chestnut extract to be used in CVD	28.5	52.4	57.9	No
	Horse chestnut extract considered effective in improving symptoms of CVD	23.8	47.6	57.9	No
	Hydroxyethylrutosides to be used in CVD	14.3	42.9	61.1	No
	Hydroxyethylrutosides considered effective in improving symptoms of CVD	23.8	42.9	42.1	No
	Red vine leaf extract to be used in CVD	23.8	42.9	26.3	No
	Red vine leaf extract considered effective in improving symptoms of CVD	23.8	38.1	21.1	No
	Hesperidin to be used in CVD	66.7	85.7	89.5	Yes
	Hesperidin considered effective in improving symptoms of CVD	62.0	85.7	73.7	Yes
	Anthocyanosides to be used in CVD	28.6	38.1	63.2	No
	Anthocyanosides considered effective in improving symptoms of CVD	28.5	42.9	42.1	No
	β-arbutin to be used in CVD	0.0	23.8	26.3	No
	β-arbutin considered effective in improving symptoms of CVD	0.0	23.8	26.3	No
	Guidelines to be updated to reflect the role of nutraceuticals in CVD treatment based on new evidence	76.2	85.7	–	Yes
Combination regimens	Combination therapy to be used in symptomatic CVD	76.1	100.0	–	Yes
	Combination therapy considered more effective than monotherapy	90.5	100.0	–	Yes
	Specific oral combination (diosmin + ruscus + melilotus + Vitis vinifera + horse chestnut extract) considered a reasonable first-line conservative option	76.2	85.7	–	Yes
Topical agents	Use topical agents in C0	28.5	19.0	22.2	No
	Use topical agents in C1–C2	42.9	66.7	78.9	Yes
	Use topical agents in C3–C6	66.6	81.0	84.2	Yes
	Topicals agents should reduce edema and hematoma	9.5	66.7	94.7	Yes
	Topicals agents should reduce itching and burning	19.0	57.1	84.2	Yes
	Topicals agents should reduce heaviness	33.3	71.4	84.2	Yes
	Topical agents should improve skin changes	0.0	61.9	73.7	Yes
	Topicals agents should have all the above effects	38.1	61.9	84.2	Yes
	Specific topical combination (diosmin + ruscus + melilotus + Vitis vinifera + horse chestnut extract) considered a reasonable option	90.5	90.5	–	Yes

CVD: chronic venous disease; CVI: chronic venous insufficiency; MPFF: micronized purified flavonoid fraction; QoL: quality of life.

## Data Availability

The data presented in this study are available on request from the corresponding author due to privacy and ethical restrictions.

## Supplementary Materials

The following supporting information can be downloaded at: https://www.mdpi.com/article/10.3390/nu17243830/s1, Table S1: Comparative overview of international guideline recommendations on venoactive nutraceuticals.

## Abbreviations

The following abbreviations are used in this manuscript:

ACR	American College of Radiology
AVF	American Venous Forum
AVLS	American Vein and Lymphatic Society
CVD	Chronic Venous Disease
CVI	Chronic Venous Insufficiency
CEAP	Clinical, Etiological, Anatomical, and Pathophysiological classification
ESVS	European Society for Vascular Surgery
IUA	International Union of Angiology
MPFF	Micronized Purified Flavonoid Fraction
PROM	Patient-Reported Outcome Measure
QoL	Quality of Life
RCT	Randomized Controlled Trial
r-VCSS	Revised Venous Clinical Severity Score
SIAPAV	Società Italiana di Angiologia e Patologia Vascolare/Italian Society of Angiology and Vascular Medicine
SVS	Society for Vascular Surgery
