# Supplementary material for: Delphi Consensus on the Role of Venoactive Nutraceuticals in the Management of Chronic Venous Disease: A Position Statement of the Italian Society of Angiology and Vascular Medicine (SIAPAV)
Source: Nutrients. 2025 Dec 7;17(24):3830. doi: 10.3390/nu17243830 (PMC12735476; doi:10.3390/nu17243830)
Supplement: Supplementary file 1 [file nutrients-17-03830-s001.zip › nutrients-3990489-supplementary.pdf]

Table S1. Comparative overview of international guideline recommendations on venoactive nutraceuticals.

Domain	Statement	Agreement (%)	IUA 2018	ESVS 2022	SVS/AVF/AVLS 2023
Classification and assessment	Use of CEAP classification in routine CVD care	Yes (90.5)	Reported but not formally recommended	Recommended (Class I, Level C)	Recommended
	Adoption of 2020 revision of the CEAP classification	Yes (81.0)	Not reported	Recommended (Class I, Level C)	Recommended
	Clinical distinction between CVD (C0–C2) from CVI (C3–C6)	Yes (100.0)	Reported but not formally recommended	Reported but not formally recommended	Reported but not formally recommended
	Classification of patients as symptomatic vs asymptomatic	Yes (81.0)	Reported but not formally recommended	Recommended (Class I, Level C)	Recommended
	Use of validated QoL questionnaires in routine practice	No (47.4)	Not reported	Use of r-VCSS suggested (Class IIa, Level C)	Use of r-VCSS recommended
Indications across CEAP classes	Use of venoactive nutraceuticals in C0	Yes (84.2)	Generically reported in symptomatic CVD with no restriction on CEAP class. Strong (1B) to weak (2C) recommendation depending on the agent	Generically suggested (Class IIa, Level A) in symptomatic CVD with no restriction on CEAP class	Not reported
	Use of venoactive nutraceuticals in C1–C2	Yes (95.2)	Generically reported in symptomatic CVD with no restriction on CEAP class. Strong (1B) to weak (2C) recommendation depending on the agent	Generically suggested (Class IIa, Level A) in symptomatic CVD with no restriction on CEAP class	Generically suggested (2B to 2C, depending on the agent) in symptomatic patients with varicose veins

	Use of venoactive nutraceuticals in in C3–C6	Yes (90.5)	Generically reported in symptomatic CVD with no restriction on CEAP class. Strong (1B) to weak (2C) recommendation depending on the agent	Generically suggested (Class IIa, Level A) in symptomatic CVD with no restriction on CEAP class	Not reported
	Use of guideline-endorsed venoactives in symptomatic CVD	Yes (90.5)	Strong (1B) to weak (2C) recommendation provided depending on the agent	Not reported	Weak recommendation (2B to 2C, depending on the agent)
Single-agent selection	MPFF to be used in CVD	Yes (90.5)	Strongly recommended in symptomatic CVD (1B)	Not reported	Suggested in symptomatic patients with varicose veins (2B)
	MPFF considered effective in improving symptoms of CVD	Yes (85.7)	Reported (moderate evidence)	Not reported	Reported (moderate evidence)
	Diosmin to be used in CVD	Yes (95.2)	Weakly recommended in symptomatic CVD (2C)	Not reported	Not reported
	Diosmin considered effective in improving symptoms of CVD	Yes (100.0)	Reported (weak evidence)	Not reported	Not reported
	Ruscus extract to be used in CVD	No (52.6)	Weakly recommended in symptomatic CVD (2B)	Not reported	Suggested in symptomatic patients with varicose veins (2B)
	Ruscus extract considered effective in improving symptoms of CVD	No (52.6)	Reported (moderate evidence)	Not reported	Reported (moderate evidence)
	Horse chestnut extract to be used in CVD	No (57.9)	Weakly recommended in symptomatic CVD (2B)	Not reported	Suggested in symptomatic

					patients with varicose veins (2C)
	Horse chestnut extract considered effective in improving symptoms of CVD	No (57.9)	Reported (moderate evidence)	Not reported	Reported (weak evidence)
	Hydroxyethylrutosides to be used in CVD	No (61.1)	Weakly recommended in symptomatic CVD (2B)	Not reported	Suggested in symptomatic patients with varicose veins (2C)
	Hydroxyethylrutosides considered effective in improving symptoms of CVD	No (42.1)	Reported (moderate evidence)	Not reported	Reported (weak evidence)
	Red vine leaf extract to be used in CVD	No (26.3)	Weakly recommended in symptomatic CVD (2B)	Not reported	Suggested in symptomatic patients with varicose veins (2C)
	Red vine leaf extract considered effective in improving symptoms of CVD	No (21.1)	Reported (moderate evidence)	Not reported	Reported (weak evidence)
	Hesperidin to be used in CVD	Yes (89.5)	Not reported	Not reported	Not reported
	Hesperidin considered effective in improving symptoms of CVD	Yes (73.7)	Not reported	Not reported	Not reported
	Anthocyanosides to be used in CVD	No (63.2)	Not reported	Not reported	Not reported
	Anthocyanosides considered effective in improving symptoms of CVD	No (42.1)	Not reported	Not reported	Not reported
	β-arbutin to be used in CVD	No (26.3)	Not reported	Not reported	Not reported
	β-arbutin considered effective in improving symptoms of CVD	No (26.3)	Not reported	Not reported	Not reported
	Guidelines to be updated to reflect the role of nutraceuticals	Yes (85.7)	Not reported	Not reported	Not reported

	in CVD treatment based on new evidence				
Combination regimens	Combination therapy to be used in symptomatic CVD	Yes (100.0)	Not reported	Not reported	Not reported
	Combination therapy considered more effective than monotherapy	Yes (100.0)	Not reported	Not reported	Not reported
	Specific oral combination (diosmin + ruscus + melilotus + vitis vinifera + horse chestnut extract) considered a reasonable first-line conservative option	Yes (85.7)	Not reported	Not reported	Not reported
Topical agents	Use topical agents in C0	No (22.2)	Not reported	Not reported	Not reported
	Use topical agents in C1–C2	Yes (78.9)	Not reported	Not reported	Not reported
	Use topical agents in C3–C6	Yes (84.2)	Not reported	Not reported	Not reported
	Topicals agents should reduce edema and hematoma	Yes (94.7)	Not reported	Not reported	Not reported
	Topicals agents should reduce itching and burning	Yes (84.2)	Not reported	Not reported	Not reported
	Topicals agents should reduce heaviness	Yes (84.2)	Not reported	Not reported	Not reported
	Topical agents should improve skin changes	Yes (73.7)	Not reported	Not reported	Not reported
	Topicals agents should have all the above effects	Yes (84.2)	Not reported	Not reported	Not reported
	Specific topical combination (diosmin + ruscus + melilotus + vitis vinifera + horse chestnut extract) considered a reasonable option	Yes (90.5)	Not reported	Not reported	Not reported

CVD: chronic venous disease; CVI: chronic venous insufficiency; MPFF: micronized purified flavo-noid fraction; QoL: quality of life.
